# Coordinated Arp2/3-mediated actin branching and myosin-dependent force generation in membrane–cytoskeleton remodeling

**DOI:** 10.3389/fphys.2026.1919458

**Published:** 2026-09-14

**Authors:** Chunzhen Jiang, Yifan Li, Jianghong He

**Affiliations:** 1 Key Laboratory of Neuroregeneration of the Ministry of Education, Collaborative Innovation Center for Neuroregeneration of Jiangsu Province, Nantong University, Nantong, Jiangsu, China; 2 Department of Orthopedics, Affiliated Fuyang People’s Hospital of Anhui Medical University, Fuyang, Anhui, China

**Keywords:** ARP2/3 complex, branched actin, endocytosis, mechanotransduction, membrane-cytoskeleton, myosin

## Abstract

The Arp2/3 (Actin-Related Protein 2/3) complex nucleates branched actin networks, while myosin motors generate force and reorganize actin through ATP-dependent motor activity. Their coordination is central to membrane remodeling, but the underlying mechanisms differ substantially among cellular processes and organisms. In this review, we distinguish direct molecular coupling from indirect mechanical coupling and signaling cross-talk. We also separate mechanisms established in fungal systems from those supported in mammalian cells. Across endocytosis, migration, secretion, membrane repair, and membrane fusion, Arp2/3 primarily determines where and how branched networks are nucleated, whereas polymerizing actin filaments generate protrusive or invaginating force. Distinct myosin isoforms then contribute membrane anchoring, contractile tension, network remodeling, or cargo transport. We further discuss membrane and cortical tension, retrograde flow, actin turnover, mTOR and Rho signaling, disease relevance, and unresolved questions. This mechanistic framework clarifies which aspects of Arp2/3–myosin coordination are experimentally demonstrated and which remain context-dependent models.

## 1 Introduction

The plasma membrane and cortical cytoskeleton form a mechanically coupled interface that integrates membrane organization with actin remodeling (Di Giaimo et al., 2020; Yan et al., 2024). Membrane tension, curvature, and receptor organization provide spatial constraints, while the cortical actin network provides structural support and rapid force transmission. This interface is therefore the relevant physical setting for examining how Arp2/3-dependent network assembly and myosin-dependent mechanics are coordinated.

Within this interface, the Arp2/3 complex is a major regulator of branched actin architecture. It nucleates daughter filaments from pre-existing actin and supports rapid remodeling of lamellipodia, cortical actin, endocytic networks, vesicle-associated actin, and pathogen-associated actin structures (Mullins et al., 1998; May, 2001; Goley and Welch, 2006; Zimmet et al., 2020; Carlson and Nolen, 2026). Arp2/3 determines branch geometry, whereas polymerization at filament barbed ends supplies protrusive or invaginating force. Efficient activation requires nucleation-promoting factors (NPFs), a mother filament, and context-specific membrane signals. Structural work supports two NPF-binding sites and shows that selected WASP-family proteins can couple Arp2/3 binding to actin-monomer delivery (Zimmet et al., 2020; Carlson and Nolen, 2026). Arp2/3 is therefore an architectural nucleator embedded in a regulated membrane-associated system, not an autonomous force generator.

Arp2/3 and myosin influence the same membrane-associated actin systems, but their relationship is not a single universal form of synergy. Depending on context, they can be connected by direct or scaffold-mediated interactions, force transmission through a shared actin network, or reciprocal signaling between protrusive and contractile modules. At adherens junctions, actomyosin-dependent assembly of mechanosensitive machinery can trigger local actin polymerization and organization (Favarin et al., 2026). These mechanisms must be distinguished because direct myosin-I-dependent Arp2/3 activation in fungi cannot be generalized to mammalian cells, and myosin-II usually acts through spatially separated contractile networks rather than direct binding to Arp2/3.

This review therefore focuses on the molecular basis of Arp2/3–myosin coordination. We first compare the structural and regulatory properties of Arp2/3 and major myosin isoforms. We then define mechanistic modes of coordination and use this framework to interpret endocytosis, migration, secretion, membrane repair, and membrane fusion. We explicitly separate organism-specific mechanisms, evaluate the strength of evidence for proposed interactions, and identify unresolved questions with particular attention to biophysical regulation, mTOR signaling, and human disease.

## 2 Structural basis and molecular regulation mechanisms of the Arp2/3 complex

### 2.1 Composition, structural dynamics, and activation mechanism of the Arp2/3 complex

The Arp2/3 complex is a seven-subunit actin nucleator composed of Arp2, Arp3, and ARPC1-5 (**Table 1**). Arp2 and Arp3 form an actin-like nucleation template, whereas ARPC subunits stabilize the complex and provide contacts with the mother filament and branch junction (**Figure 1**) (Mullins et al., 1998; Zimmet et al., 2020). Activation reorganizes Arp2 and Arp3 into a short-pitch arrangement that resembles consecutive subunits in an actin filament. NPF-bound structural studies define the activated complex, while CK-666 and CK-869 inhibit nucleation by stabilizing or inducing inactive conformations (Hetrick et al., 2013; Zimmet et al., 2020; Cao et al., 2024). This structural cycle explains how branch formation can be rapidly switched on and off without assigning a single exclusive function to each small subunit.

**Table 1:** Composition of the Arp2/3 complex.

Subunit	Approximate molecular mass	Evidence-based functional description
Arp2	~45 kDa	Actin-related subunit that joins Arp3 to form the short-pitch nucleation template (Mullins et al., 1998; Zimmet et al., 2020).
Arp3	~47 kDa	Actin-related subunit that completes the nucleation template and contains an NPF-binding site (Zimmet et al., 2020).
ARPC1/p41	~41 kDa	WD-repeat subunit that contributes regulatory contacts and participates in the Arp2-ARPC1 NPF-binding region (Zimmet et al., 2020).
ARPC2/p34	~34 kDa	Core structural subunit that contributes to the integrity of the Arp2/3 complex (Mullins et al., 1998; Zimmet et al., 2020).
ARPC3/p21	~21 kDa	Small subunit with stabilizing and regulatory roles; its contribution should not be reduced to a single exclusive contact.
ARPC4/p20	~20 kDa	Core scaffold subunit that supports the structural organization of the Arp2/3 complex (Mullins et al., 1998; Zimmet et al., 2020).
ARPC5/p16	~16 kDa	Small, isoform-dependent subunit that modulates complex activity and cellular function; ARPC5 deficiency has immune consequences (Cao et al., 2024; Vasconcellos et al., 2025).

**Figure 1:**
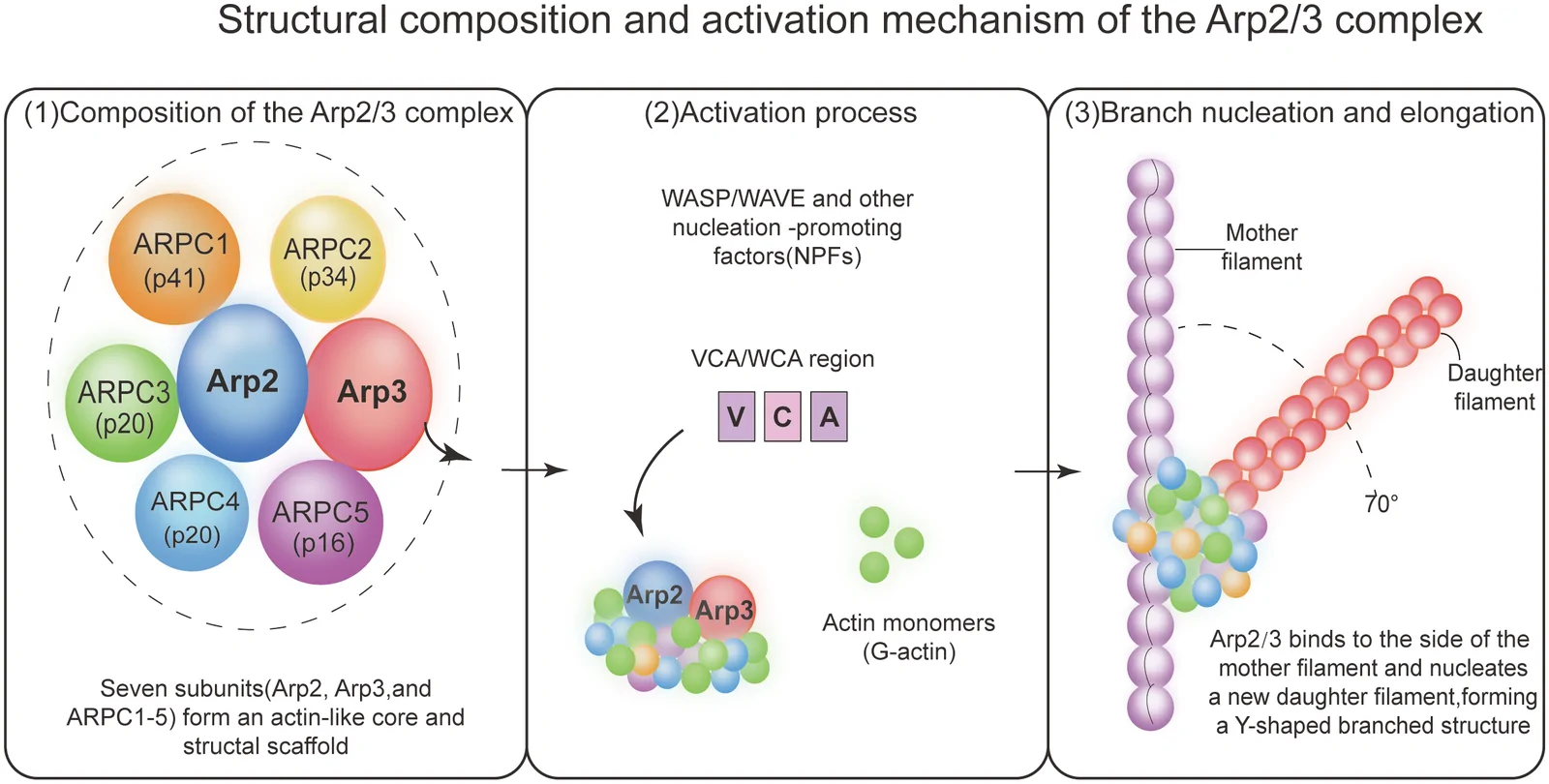
Structural composition and activation mechanism of the Arp2/3 complex. (1) The seven-subunit complex comprises Arp2, Arp3, and ARPC1-5, which form an actin-like nucleation core and structural scaffold. (2) WASP/WAVE-family nucleation-promoting factors engage Arp2/3 through WCA/VCA regions, promote actin-monomer recruitment, and induce the activating conformational rearrangement. (3) Activated Arp2/3 binds the side of a mother filament and nucleates a daughter filament at an angle of approximately 70 degrees, generating a Y-shaped branch.

### 2.2 Key regulatory factors and collaborative activation networks

Because isolated Arp2/3 has weak nucleation activity, efficient branching requires NPFs and a pre-existing mother filament (**Table 2**). WASP-family proteins provide WCA or VCA regions that bind Arp2/3 and actin monomers (Higgs and Pollard, 2001). *In vivo* work further shows that WASP-Arp2/3 activity can assemble F-actin on fused secretory vesicles (Tran et al., 2015). Structural studies identify two NPF-binding sites, one centered on Arp3 and another near Arp2-ARPC1, and show that their contributions vary among NPF families (Zimmet et al., 2020; Carlson and Nolen, 2026). The dual-site mechanism supports regulated activation, but it should not be interpreted as evidence that every NPF uses identical stoichiometry or actin-delivery requirements.

**Table 2:** The dynamic cycle regulated by the Arp2/3 complex.

Regulatory role	Mechanistic class	Factor(s)	Primary target or action	Mechanistic effect
Activation	Class I NPF	WASP/N-WASP and WAVE/SCAR	WCA/VCA regions bind Arp2/3; selected NPFs deliver actin monomers (Higgs and Pollard, 2001; Zimmet et al., 2020; Carlson and Nolen, 2026).	Promote localized branch nucleation; requirements differ among NPF families.
Activation/stabilization	Class II NPF and branch stabilizer	Cortactin	Binds Arp2/3 and branch junctions and cooperates with WASP-family proteins (Uruno et al., 2001; Fregoso et al., 2023).	Enhances nucleation in defined contexts and stabilizes newly formed branches.
Inhibition	Inhibitory NPF	Arpin	Antagonizes Arp2/3 activation by competing with activating nucleation-promoting factors (Cao and Way, 2024).	Dispensable for chemotaxis in the tested model; broader migration effects are context dependent (Dang et al., 2017).
Debranching	Branch-disassembly factors	GMF and coronins, including Coro7	Destabilize branch junctions or promote Arp2/3 release (Guo et al., 2018; Shatery Nejad et al., 2025).	Increase branch turnover and permit network remodeling.
Turnover/renewal	Branch regeneration	Nucleotide exchange on retained Arp2/3	Permits renucleation after load-induced branch dissociation (Ghasemi et al., 2024).	Supports reuse of Arp2/3 at mother filaments under defined conditions.

Arp2/3 activity extends beyond the plasma membrane; on lipid droplets, Arp2/3-dependent actin influences organelle motility and lipid synthesis (Zhao et al., 2022). At the cell cortex, N-WASP is regulated by Cdc42 and phosphoinositides, whereas WAVE-family pathways act downstream of Rac to support lamellipodial protrusion (Steffen et al., 2006; Chemeris et al., 2018). Membrane curvature can further concentrate FBP17, Cdc42, and N-WASP to initiate local nucleation (Zhu et al., 2026). These pathways enrich Arp2/3 activity at selected sites without implying that activation is absolutely confined to one membrane compartment or that Rac directly opens the WAVE regulatory complex in every context.

Live-cell imaging, biochemical reconstitution, modeling, and quantitative biophysics collectively show that cells combine membrane curvature, small-GTPase signaling, and multivalent protein interactions to position branched-actin assembly. In the FBP17-Cdc42-N-WASP pathway, curvature-dependent clustering recruits Arp2/3 and promotes local nucleation (Zhu et al., 2026). This example illustrates context-specific deployment of a conserved nucleator rather than a single universal upstream pathway.

Arp2/3 output is controlled not only by activation but also by branch stabilization, inhibition, and disassembly. Broad regulatory schemes have been reviewed previously (Chemeris et al., 2018), while direct biochemical and structural studies show that GMF-family proteins and coronins can destabilize branch junctions or promote Arp2/3 release (Guo et al., 2018; Shatery Nejad et al., 2025). Negative regulation is therefore part of the normal branch life cycle: it limits persistence, permits network turnover, and allows repeated remodeling under changing mechanical loads.

Arpin is an inhibitory regulator that antagonizes Arp2/3 activation by competing with activating nucleation-promoting factors (Cao and Way, 2024). In the system tested by Dang et al., Arpin was dispensable for chemotaxis (Dang et al., 2017), indicating that its contribution to migration is context dependent rather than universal. Coronin also participates in wound-induced actin remodeling, illustrating a physiological setting in which branch turnover is embedded in a broader repair program (Yumura et al., 2022). Cortactin acts differently: it binds branch-associated Arp2/3, stabilizes junctions, and can cooperate with WASP-family proteins to enhance nucleation under defined conditions (Uruno et al., 2001; Fregoso et al., 2023). Effective Arp2/3 function therefore depends on a regulated cycle of branch generation, stabilization, inhibition, disassembly, and renewal rather than maximal activation alone.

## 3 Functional specialization of myosin isoforms at the membrane–cytoskeleton interface

Myosins are ATP-dependent actin motors that convert nucleotide hydrolysis into movement, tension, or network reorganization (Walklate et al., 2016). Their mechanical output depends on isoform-specific motor kinetics, oligomeric state, cargo-binding regions, and cellular localization. These differences are essential for interpreting how individual myosins cooperate with Arp2/3-dependent branched actin networks.

Myosins are commonly separated into conventional myosin-II and multiple classes of unconventional myosins (Cheney and Mooseker, 1992). This classification is functionally important because different classes specialize in contractility, membrane association, cargo transport, or other forms of actin-based motility. The relevant properties of individual myosin classes are introduced below together with class-specific evidence. The term myosin is therefore avoided below when the responsible isoform is known.

### 3.1 Mechanical regulation by myosin-I motors

The myosin-I family comprises membrane-associated unconventional motors with diverse membrane-binding regions and regulatory mechanisms (Berg et al., 2001; Heissler and Sellers, 2016). Many myosin-I proteins couple the plasma membrane to cortical actin and participate in membrane deformation and endocytosis (Pedersen and Drubin, 2019). Fungal myosin-I proteins can additionally contribute nucleation-promoting activity (Willet et al., 2023). In mammalian cells, membrane-actin coupling is better established than direct activation of Arp2/3; Myo1E, for example, is recruited in a membrane-tension-dependent manner during clathrin-mediated endocytosis (Smith et al., 2026).

### 3.2 Myosin-II, myosin-VI, and myosin-XI have distinct mechanical roles

Myosin-II includes muscle and non-muscle isoforms and has established roles in contraction, adhesion, migration, and cytokinesis (Vicente-Manzanares et al., 2009; Walklate et al., 2016). Non-muscle myosin-II (NMII) assembles into bipolar filaments that generate contractile tension and organize cortical and stress-fiber actin. In migrating cells, NMII-dependent pressure and contractility can restrict the spatial domain of Rac-WAVE-Arp2/3-driven protrusions (Patel et al., 2021). Rho-family pathways coordinate Arp2/3-dependent assembly and NMII-dependent tension during epithelial self-contact fusion (Sumida and Yamada, 2015), while physical confinement can shift cells toward persistent migration (Paulson et al., 2025). These effects represent mechanical and signaling cross-talk rather than evidence that NMII directly binds or activates Arp2/3.

Myosin-VI is unusual because it moves toward actin-filament minus ends. It contributes to epididymal epithelial organization in mouse models (Richert et al., 2026), participates in membrane traffic, endocytosis, and cell migration (Buss et al., 2002), and works with branched actin during melanosomal tubule constriction and fission (Ripoll et al., 2018). These isoform-specific functions should be distinguished from fungal myosin-I-dependent Arp2/3 activation and from NMII contractility.

Myosin-XI is a plant-specific motor that supports long-range intracellular transport and actin-dependent organization. In flowering plants, class XI myosin participates in sperm nuclear migration through a pathway that is distinct from canonical Arp2/3-dependent branching (Ali et al., 2020).

Myosin-I, NMII, myosin-VI, and myosin-XI should not be treated as interchangeable motors. Myosin-I primarily links membranes and actin and can be load sensitive, and selected fungal proteins also contain Arp2/3-activating regions. NMII generates contractile tension through bipolar filaments. Myosin-VI is a minus-end-directed cargo motor involved in membrane traffic, whereas myosin-XI is plant specific. The type of Arp2/3-myosin coordination consequently depends on the myosin isoform, actin architecture, membrane geometry, and organism.

## 4 Mechanistic modes of Arp2/3–myosin coordination

### 4.1 Direct molecular coupling is context restricted

Available evidence does not support a universal direct interaction between Arp2/3 and myosin. The clearest direct or scaffold-mediated links occur in fungal endocytosis, where type-I myosins, Las17 or Wsp1, Vrp1, and associated SH3-domain proteins assemble Arp2/3-activating modules at endocytic sites (Pedersen and Drubin, 2019; Willet et al., 2023; Hill et al., 2024b). In Dictyostelium, CARMIL can connect type-I myosins with capping protein and the Arp2/3 complex (Jung et al., 2001). By contrast, mammalian myosin-I proteins are better supported as membrane–actin linkers and load-sensitive motors. Current mammalian evidence does not establish myosin-I as a direct Arp2/3 activator. Functional cooperation can therefore occur without direct biochemical activation.

### 4.2 Indirect mechanical coupling and spatial division of labor

Most coordination in migration and membrane remodeling is indirect. Arp2/3 nucleates branched filaments, while polymerization at filament barbed ends produces protrusive or invaginating force. Myosin changes how this force is transmitted by anchoring actin to membranes, producing contractile tension, remodeling network architecture, or moving cargo. In migrating cells, Arp2/3-rich and NMII-rich networks are often spatially separated at the front and rear. Their outputs are coupled through retrograde flow, membrane tension, adhesions, and Rho-family signaling rather than through a stable Arp2/3–NMII complex. Recent perturbation studies also support distinct but interacting feedback loops between branched actin and actomyosin (Kuhn et al., 2025).

### 4.3 Evidence levels for proposed coordination

To avoid interpreting association as direct mechanism, evidence is separated into four levels in this review. Direct molecular coupling requires physical interaction or reconstituted biochemical activity. Mechanical coupling refers to force transmission through a shared actin or membrane system without demonstrated protein binding. Signaling cross-talk refers to shared upstream or reciprocal regulatory pathways. Colocalization or correlated phenotypes are considered supportive evidence but are not sufficient to establish direct interaction. The term coordinated action is therefore used when experiments show functional interdependence, whereas synergy is reserved for studies that demonstrate an enhanced combined output.

These modes are not mutually exclusive. Fungal endocytosis can combine membrane anchoring, motor activity, and direct nucleation-promoting activity within one local module. Dictyostelium can use CARMIL as a scaffold between type-I myosin, capping protein, and Arp2/3. Mammalian systems more often support indirect mechanical coupling through shared actin networks and signaling. The conserved feature is functional coordination at the membrane-actin interface, whereas the molecular linkage itself is not universally conserved. Context-specific physiological outputs are summarized in **Table 3**, and the mechanistic modes and their evidence levels are compared in **Table 4**.

**Table 3:** Summary of context-specific coordination between Arp2/3 and myosin isoforms.

Physiological process	Primary Arp2/3 function	Primary myosin function	Representative outcome, model, and evidence level
Endocytosis	Nucleates branched actin; filament polymerization supports invagination.	myosin-I couples actin to membrane and becomes more important under elevated load.	Vesicle internalization; mammalian imaging/perturbation and fungal mechanistic studies (Pedersen and Drubin, 2019; Willet et al., 2023; Hill et al., 2024b; Smith et al., 2026).
Cell migration	Supports leading-edge branched networks and protrusion.	NMII generates rear and cortical tension, retrograde flow, and adhesion remodeling.	Directional migration; mammalian cells, amoebae, and growth cones; mechanical/signaling evidence (Yang et al., 2012; Patel et al., 2021; Kim and Kim, 2025; Kuhn et al., 2025).
Secretion	Organizes vesicle-associated actin and supports post-fusion cargo expulsion and vesicular-membrane integration.	Myo1b cooperates with F-actin and Arp2/3 during secretory-granule biogenesis at the trans-Golgi network; NMII contributes late contraction; myosin-VI supports selected fission events.	Drosophila and mammalian cell studies; temporal and functional coordination (Tran et al., 2015; Delestre-Delacour et al., 2017; Biton et al., 2023).
Membrane repair	Contributes to wound-induced cortical actin assembly.	myosin-I can anchor membrane-actin modules; NMII involvement is model dependent.	Dictyostelium wound repair; recruitment and loss-of-function evidence (Yumura et al., 2022).
Virus entry and trafficking	Contributes to early endocytic organization and actin-tail propulsion.	myosin-IIB supports late uncoating; myosin-VI can mediate an alternative actin-based transport route.	Influenza A virus; stage-specific imaging and perturbation evidence (Du et al., 2024; Jiang et al., 2025).

**Table 4:** Mechanistic modes of Arp2/3 and myosin coordination across systems.

Mode	Representative system	Molecular basis	Evidence and interpretation
Direct/modular activation	Budding-yeast endocytosis	Type-I myosin motor and actin-assembly activation activities are separable but locally coordinated (Hill et al., 2024b).	Genetic and cellular evidence; organism-specific and not established for mammalian myosin-I.
Recruitment control	Fission-yeast endocytosis	Ank1 restricts nonspecific Myo1 membrane binding and spatially limits endocytic activity (Willet et al., 2023).	Genetic and imaging evidence for regulatory coupling; not proof of a conserved mammalian activation mechanism.
Structural anchoring	Mammalian clathrin-mediated endocytosis	Myo1E couples cortical actin to membrane and responds to membrane tension (Smith et al., 2026).	Imaging and perturbation support load-dependent mechanical coordination, not direct Arp2/3 activation.
CARMIL-mediated bridging	Dictyostelium discoideum	CARMIL links type-I myosin, capping protein, and Arp2/3 through distinct interactions (Jung et al., 2001).	Scaffold-mediated molecular coupling supported by biochemical interaction data.
Mechanical/signaling coupling	Migrating cells and neuronal growth cones	Arp2/3-dependent protrusion and NMII-dependent contractility interact through actin flow, pressure, adhesion, and Rho signaling (Yang et al., 2012; Patel et al., 2021).	Spatially separated modules; cell perturbation supports mechanical cross-talk rather than stable binding.
Reconstituted mechanical synergy	Actin-comet system	myosin-I remodels Arp2/3-dependent branched networks and increases pushing efficiency (Xu et al., 2024).	Purified reconstitution provides strong evidence for context-dependent synergy in the tested system.

## 5 Context-specific implementation of the common mechanisms

### 5.1 Endocytosis

#### 5.1.1 Mammalian clathrin-mediated endocytosis

Clathrin-mediated endocytosis requires progressive membrane bending, invagination, and vesicle internalization. Arp2/3 nucleates a branched actin network at selected endocytic sites, but polymerizing filaments generate the force that opposes membrane tension. Mammalian myosin-I can couple this network to the plasma membrane and add motor-dependent mechanical output. Arp2/3 therefore controls nucleation and architecture, whereas actin polymerization and myosin-I-dependent coupling determine how force is transmitted to the membrane (Smith et al., 2026).

At mammalian endocytic sites, WASP-family NPFs activate Arp2/3, while myosin-I is best interpreted as a membrane-actin linker and load-sensitive motor rather than a general Arp2/3 activator. Work in yeast established that type-I myosins can anchor actin assembly to the plasma membrane (Pedersen and Drubin, 2019), and mammalian Myo1E imaging supports load-dependent coordination with Arp2/3 recruitment (Smith et al., 2026). The acidic-domain activation mechanism described for fungal myosin-I should not be generalized to mammalian isoforms without direct biochemical evidence.

A reconstituted actin-comet system showed that myosin-I can increase the pushing capacity of Arp2/3-dependent branched networks by remodeling network organization (Xu et al., 2024). Related reconstitution demonstrates that cross-linking proteins can further alter branched-network organization and force generation (Hill et al., 2024a). Together, these studies provide direct evidence for context-dependent mechanical synergy in defined systems, but they do not imply that myosin-I universally activates Arp2/3 in cells.

In human induced pluripotent stem cells, Myo1E recruitment increases when membrane tension is elevated (Smith et al., 2026). Loss of Myo1E alters the timing and amount of Arp2/3 accumulation at endocytic sites and becomes particularly detrimental under high load. These observations support load-dependent coordination between Myo1E and branched actin assembly. They are consistent with indirect recruitment and force coupling, but they do not establish direct biochemical activation of Arp2/3 by mammalian Myo1E.

#### 5.1.2 Fungal endocytosis

In budding and fission yeast, type-I myosins are integral components of endocytic actin-assembly modules. They anchor actin-regulatory factors at the plasma membrane and couple actin polymerization to membrane invagination (Pedersen and Drubin, 2019; Willet et al., 2023). These fungal systems provide the strongest evidence that myosin-I can combine motor, membrane-binding, and nucleation-promoting functions within the same endocytic machinery.

During budding-yeast endocytosis, Arp2/3-dependent filament polymerization helps overcome the mechanical load imposed by turgor pressure. Type-I myosins contribute membrane anchoring and motor activity, and their actin-assembly activation function can be experimentally separated from motor function (Pedersen and Drubin, 2019; Hill et al., 2024b). This organism-specific combination of activities should not be extrapolated to mammalian myosin-I proteins.

Fungal systems also demonstrate that recruitment and activation are distinct regulatory steps. In fission yeast, Ank1 restricts nonspecific Myo1 membrane association and helps confine endocytic activity to appropriate cortical sites (Willet et al., 2023). In budding yeast, myosin-I motor and actin-assembly activation activities are modular but must be spatially coordinated (Hill et al., 2024b). These findings support multifunctional local coupling rather than a universal one-step activation pathway.

Motor activity and the TH1 membrane-binding region support force transmission and membrane attachment, whereas nucleation-promoting activity is mechanistically separable (Pedersen and Drubin, 2019; Hill et al., 2024b). Type-I myosin can therefore remain important when one biochemical contribution is reduced, and phenotypes should not automatically be assigned to direct Arp2/3 activation.

The membrane recruitment of fungal myosin-I is also regulated. Ank1 limits nonspecific myosin-I membrane association and thereby helps restrict endocytic activity to appropriate cortical sites (Willet et al., 2023). Although a human homolog, OSTF1, exists, the available evidence does not justify assuming that mammalian cells use the same myosin-I-dependent Arp2/3 activation mechanism.

#### 5.1.3 Influenza virus endocytic trafficking

During influenza A virus entry, endosomal trafficking, and uncoating, Arp2/3-dependent actin remodeling and myosin-IIB-dependent contractility can contribute at different stages. These roles are temporally separated and should not be interpreted as evidence for a stable Arp2/3–myosin-IIB complex.

Early after endocytic entry, branched actin organization can influence vesicle movement and access to downstream trafficking routes. Arp2/3 contributes by nucleating the relevant actin network, while filament polymerization and other motors generate movement. Adducin-1 regulates branched actin density around virus-containing endosomes and thereby influences trafficking (Jiang et al., 2025).

At a later stage, myosin-IIB-dependent actomyosin tension contributes to influenza virus uncoating. Adducin-1 promotes RhoA and myosin light-chain signaling, and reduced Adducin-1 lowers myosin-IIB engagement with actin and decreases the mechanical output required for efficient capsid disassembly (Jiang et al., 2025). This is a contractility-dependent mechanism that is distinct from the earlier Arp2/3-dependent actin-remodeling step.

The available data therefore support stage-specific coordination. Arp2/3-dependent branched actin contributes mainly to early endocytic organization and trafficking, whereas myosin-IIB is more directly involved in late mechanical uncoating (Jiang et al., 2025). Their regulation by Adducin-1 provides pathway-level coordination without demonstrating direct Arp2/3–myosin-IIB binding.

Single-virus tracking identifies three routes by which influenza A virus reaches microtubules after endocytosis: myosin-VI-dependent transport along actin, Arp2/3-dependent actin-tail propulsion, and a direct non-actin route (Du et al., 2024). The direct route was fastest and predominant under the tested conditions, whereas actin-tail propulsion became more prominent at high viral load. These results broaden the trafficking model but do not establish direct coupling between myosin-VI and Arp2/3.

### 5.2 Cell migration

#### 5.2.1 Arp2/3 and myosin-I at protrusive regions

Arp2/3 and myosin-I can occupy overlapping membrane-associated actin regions, but the consequences depend on cell type. Arp2/3-dependent polymerization establishes protrusive branched networks, while myosin-I can couple these networks to membranes and remodel their organization. Evidence for an increase in combined pushing force is strongest in reconstituted systems rather than in all migrating cells.

In an *in vitro* actin-comet model, myosin-I enhanced the pushing force of Arp2/3-dependent branched actin networks (Xu et al., 2024). Arp2/3 established the branched architecture, filament polymerization produced the protrusive force, and myosin-I motor activity altered network density and symmetry. This experiment therefore supports true mechanical synergy in a defined system because the combined components produced a greater mechanical output than the branched network alone. The result should not be generalized to every lamellipodium without cell-specific evidence.

Fungal studies provide a different mechanism. In budding yeast, the membrane-binding, motor, and nucleation-promoting activities of type-I myosin can be experimentally separated, yet they must be recruited to the same endocytic region for normal function (Hill et al., 2024b). This modular organization demonstrates local functional coupling, but it is specific to fungal myosin-I proteins that contain nucleation-promoting regions and is not a general property of mammalian myosin-I.

CARMIL provides another form of coupling that does not require direct myosin-I-dependent activation of Arp2/3. CARMIL regulation of capping protein can influence actin assembly at protrusive edges (Fujiwara et al., 2014). In Dictyostelium discoideum, CARMIL also links type-I myosins, the Arp2/3 complex, and capping protein through distinct binding interactions (Jung et al., 2001). This is best classified as scaffold-mediated coordination of membrane-associated actin regulators.

Studies of non-monotonic spreading in Dictyostelium discoideum demonstrate rapid reorganization of cortical actin during changes in cell shape (Heinrich et al., 2008). However, these observations do not by themselves establish a direct myosin-I–Arp2/3 interaction. They are therefore interpreted here as evidence for coordinated cytoskeletal dynamics rather than direct molecular synergy.

#### 5.2.2 Arp2/3 and myosin-II in front–rear mechanics

During migration, Arp2/3-dependent protrusion and NMII-dependent contraction are mechanically and spatially coupled. Arp2/3-rich networks dominate at the leading edge, whereas NMII-rich networks generate contractile stress behind the protrusive zone and at the rear. This front-rear push-pull organization is summarized in **Figure 2** and is better interpreted as mechanical and signaling coordination than as direct molecular synergy.

**Figure 2:**
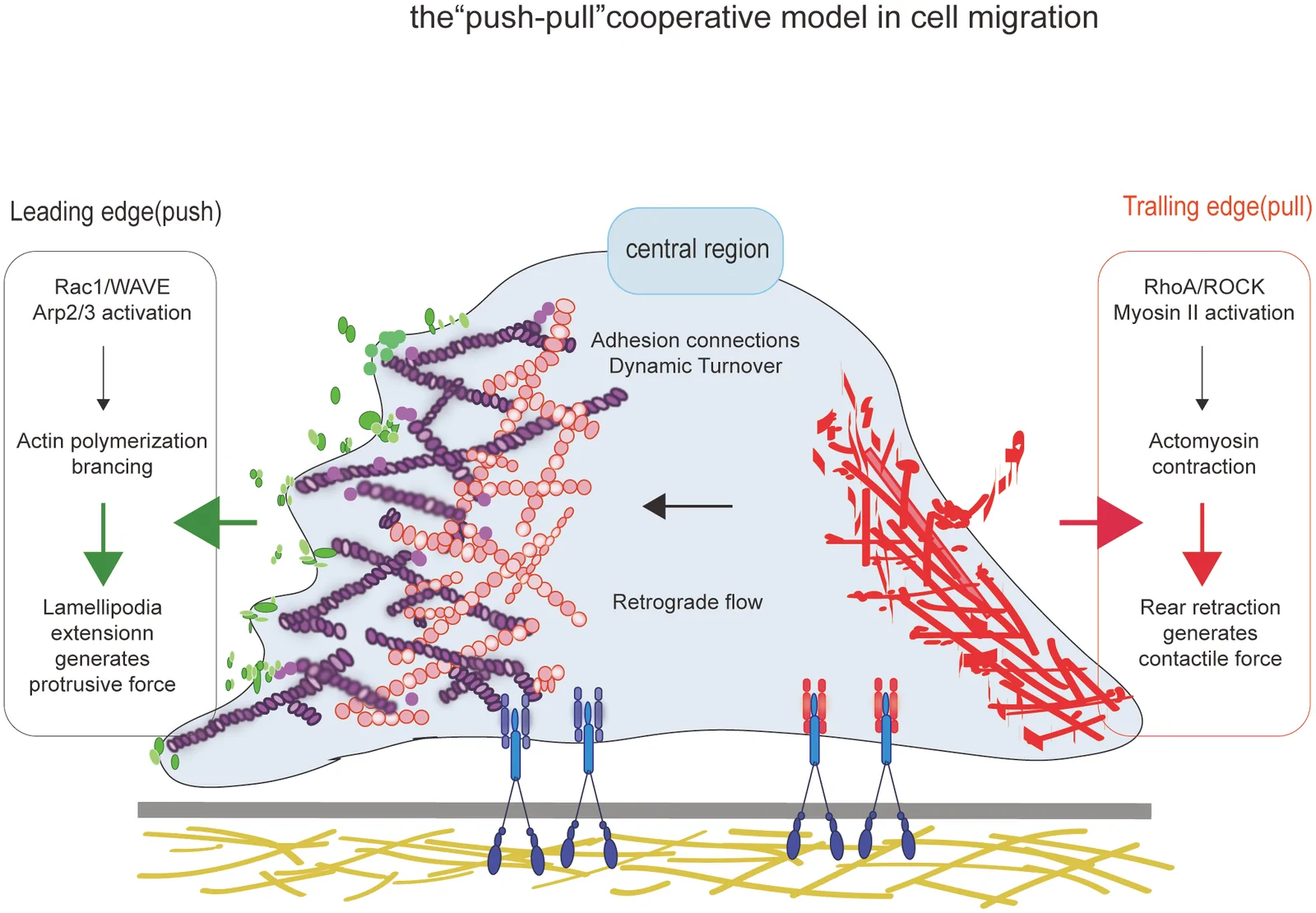
The push-pull cooperative model in cell migration. At the leading edge, Rac1-WAVE-Arp2/3 signaling promotes branched-actin polymerization and lamellipodial protrusion (push). Adhesion turnover and retrograde flow mechanically connect this protrusive network to the central region. At the trailing edge, RhoA-ROCK signaling activates NMII-dependent actomyosin contraction and rear retraction (pull). The diagram represents spatial mechanical and signaling coordination rather than direct binding between Arp2/3 and NMII.

Agent-based modeling of lamellipodia illustrates how these spatially separated modules can maintain a dynamic steady state (Kim and Kim, 2025). In this model, Arp2/3-mediated nucleation supplies new branched filaments at the front, while motor activity, crosslinking, and focal adhesions regulate network organization, retrograde flow, and force transmission. Adhesion and substrate mechanics modulate the resulting force balance. Because this study is computational, its proposed effects on filament depolymerization should be treated as a model prediction rather than direct evidence that NMII mechanically debranches Arp2/3 junctions.

Cell-migration experiments show reciprocal mechanical regulation between Arp2/3-dependent protrusion and NMII contractility. Arp2/3 inhibition can increase intracellular pressure and shift cells toward a more contractile state, whereas NMII activity can restrict lamellipodial persistence (Patel et al., 2021). Rho-family partitioning at epithelial self-contacts provides a related example in which Rac1/Cdc42-Arp2/3 and RhoA-NMII modules converge on membrane remodeling (Sumida and Yamada, 2015). These findings support signaling and mechanical cross-talk, not direct Arp2/3-NMII binding.

Extracellular geometry further changes this balance. Physical confinement and phagocytic uptake can induce persistent migration (Paulson et al., 2025), while aligned collagen separates the contributions of traction magnitude, traction dynamics, Arp2/3, NMII, and formins (Foroozandehfar et al., 2025; Niraula et al., 2025). At adherens junctions, actomyosin-dependent assembly of mechanosensitive machinery can also trigger local actin polymerization and organization (Favarin et al., 2026). Migration therefore cannot be reduced to a two-component Arp2/3-myosin system.

In amoeboid migration, branched and linear actin networks contribute to different aspects of protrusion and polarity. Arp2/3-dependent actin assembly supports protrusive activity, while myosin-II stabilizes the rear and lateral cortex and suppresses competing protrusions (van Haastert and Kortholt, 2026). This spatial opposition can produce effective directional persistence, but the underlying network also includes formins and multiple signaling pathways.

In Dictyostelium, localized Arp2/3-dependent actin assembly at the leading edge and myosin-II-dependent cortical contractility at the rear contribute to front–back polarity. Ras, Rap1, cGMP, and related signaling systems regulate these modules over different time scales (van Haastert, 2020). The resulting polarity is an emergent property of distributed feedback rather than a direct Arp2/3–myosin-II complex.

Neuronal growth cones provide direct evidence that Arp2/3-dependent networks can constrain NMII-driven retrograde actin flow (Yang et al., 2012). CK666 reduces leading-edge branched actin and increases retrograde flow when NMII remains active, whereas combined NMII inhibition changes this response. These results show mechanical coupling between the two systems through the actin network. They do not require direct binding between Arp2/3 and NMII.

### 5.3 Vesicle transport and secretion

In regulated exocytosis, Arp2/3 and different myosin isoforms act at distinct stages and in different cell types. Arp2/3-dependent branched actin can organize vesicle-associated actin coats, Myo1b can couple actin assembly to trans-Golgi membranes, NMII can contribute contractile force during late exocytosis, and myosin-VI can support membrane-tubule remodeling. These mechanisms should not be merged into a single generic myosin pathway.

Live imaging of Drosophila salivary glands shows that Arp2/3 and WASP are required for F-actin assembly on fused secretory vesicles (Tran et al., 2015). Polymerization of this Arp2/3-nucleated network is proposed to support cargo expulsion and integration of the vesicular membrane into the apical plasma membrane after fusion. NMII may add contractile force during later stages. The sequential timing supports functional coordination, but it does not demonstrate direct Arp2/3–NMII binding.

During the early stages of secretory-vesicle biogenesis at the trans-Golgi network (TGN), Myo1b, F-actin, and the Arp2/3 complex act together to regulate actin-network assembly and membrane remodeling. The Arp2/3 complex promotes actin nucleation and branching, whereas Myo1b, F-actin, and Arp2/3 functionally cooperate in secretory-granule biogenesis and Golgi-region actin recruitment (Delestre-Delacour et al., 2017). This early-stage cooperation is distinct from the vesicle compression and fusion-pore contraction processes involving myosin II during late exocytosis.

During exocytosis of large secretory vesicles in Drosophila larval salivary glands, Arp2/3-dependent actin assembly and NMII-dependent contraction have separable temporal functions (Biton et al., 2023). Branched actin supports fusion-pore expansion and stabilization, while NMII contributes to later constriction and content expulsion. This sequence illustrates temporal coordination of polymerization-based and contractile mechanics rather than a single shared force-generating mechanism.

Myosin-VI differs from most other myosins because it moves toward actin filament minus ends and participates in endocytosis, secretion, and cell migration (Buss et al., 2002). During melanosomal tubule-carrier fission, myosin-VI functions in the same remodeling process as Arp2/3-dependent branched actin, but this relationship is distinct from both fungal myosin-I activation and NMII contractility.

Super-resolution imaging showed that Cortactin, the p34 subunit of Arp2/3, and myosin-VI are enriched on melanosomal tubules. Genetic and pharmacological perturbations indicate that branched actin and myosin-VI are both required for efficient tubule constriction and fission (Ripoll et al., 2018). A conservative interpretation is that Arp2/3 establishes the branched actin architecture on which myosin-VI acts mechanically. Colocalization and mutual functional dependence do not, by themselves, demonstrate a stable direct Arp2/3–myosin-VI complex.

### 5.4 Membrane repair and fusion

#### 5.4.1 Cell membrane wound repair

Rapid repair of plasma-membrane wounds is essential for cell survival. In Dictyostelium, actin transiently accumulates at wounds, and failure of this response compromises closure (Yumura et al., 2022). The responsible machinery includes Arp2/3, formins, myosin isoforms, and multiple signaling pathways, so actin accumulation alone should not be equated with a single force-generating mechanism.

In Dictyostelium discoideum, Arp3 transiently accumulates at cell wounds before the major actin response (Yumura et al., 2022). Rac, WASP, formins, Arp2/3, profilin, and coronin participate in the subsequent actin remodeling. These findings place Arp2/3 within an early wound-induced assembly program, but the study does not establish that Arp2/3 alone generates the mechanical force for closure.

Myosin-I isoforms MyoB and MyoC also accumulate at Dictyostelium wound sites, with MyoB appearing before bulk actin accumulation. This timing is consistent with a membrane-anchoring or organizing function. In contrast, myosin-II transiently decreases at these wounds and is not essential for closure in this system (Yumura et al., 2022). The mechanism therefore differs from large-cell wound repair models such as Xenopus oocytes, where myosin-II-dependent contractile structures can contribute to closure.

The Dictyostelium wound response also includes TORC2, the Elmo/Dock complex, phosphoinositide signaling, phospholipase A2, and calmodulin (Yumura et al., 2022). This broad regulatory network illustrates why transient co-recruitment of Arp2/3 and myosins should not be interpreted as a self-contained two-protein mechanism. Instead, wound repair emerges from coupled signaling, actin assembly, membrane attachment, and context-dependent motor activity.

#### 5.4.2 Self-contact membrane fusion

During self-contact-induced membrane fusion in epithelial cells, Arp2/3-dependent actin assembly and NMII-dependent contractility act as downstream effectors of Rho-family signaling. Their contributions are complementary but mechanistically distinct.

Arp2/3 promotes localized branched actin assembly near self-contact sites, while NMII provides contractile tension through the RhoA–ROCK pathway. Rac1 and Cdc42 contribute to the protrusive actin program, and RhoA regulates contractility (Sumida and Yamada, 2015). Force generated by polymerization of Arp2/3-nucleated actin and NMII-dependent tension can together change membrane geometry and adhesion organization. The evidence supports pathway-level mechanical coordination, but it does not establish a direct molecular interaction between Arp2/3 and NMII.

## 6 Biophysical determinants of Arp2/3–myosin coordination

Membrane tension defines the mechanical load against which actin polymerization and membrane-associated myosins operate. Arp2/3 controls branch nucleation and network geometry, but the mechanical work that deforms a membrane is produced by filament polymerization and transmitted through membrane attachment sites. In mammalian clathrin-mediated endocytosis, elevated membrane tension increases the requirement for Myo1E and changes Arp2/3 accumulation at endocytic sites (Smith et al., 2026). Thus, membrane tension can alter the requirement for myosin-I without implying that myosin-I directly activates Arp2/3.

Cortical tension and retrograde flow are particularly important for Arp2/3–NMII coordination. NMII contractility can accelerate or redirect actin flow, while Arp2/3-dependent polymerization replenishes the leading-edge network (Yang et al., 2012; Kim and Kim, 2025). The effect of myosin-generated tension on branch lifetime remains context dependent. Current data support altered network turnover and compression, but they do not establish a universal mechanism in which NMII directly removes Arp2/3 branch junctions.

A quantitative description of this system therefore requires measurements of branch density and lifetime, filament turnover, retrograde-flow velocity, membrane and cortical tension, traction stress, and network elasticity. Reconstitution, force-sensitive assays, and optogenetic control are beginning to connect these observables (Hill et al., 2024a; Xu et al., 2024; Niraula et al., 2025; Yamamoto and Miyazaki, 2025). Recent work also shows that protrusive activity can transmit membrane tension over long distances and engage mTORC2-dependent Rho signaling, providing a mechanochemical route from a protrusive front to a contractile rear (De Belly et al., 2026). These approaches can help distinguish causal force transmission from correlated changes in cytoskeletal composition.

### 6.1 Quantitative biophysical scales

Recent single-branch experiments provide a useful force and kinetic scale. In purified Arp2/3 networks, controlled loading at approximately 1 pN accelerated branch dissociation, while the Arp2/3 complex often remained on the mother filament and could nucleate a new branch after nucleotide exchange (Ghasemi et al., 2024). In the same study, the dissociation rate of Arp2/3 remaining on the mother filament was 0.66 ± 0.16 s−1 in the ADP state and 0.57 ± 0.22 s−1 in the ATP-containing condition. These values show that branch stability and renewal can be quantified under defined load, but they should not be treated as universal cellular branch lifetimes. Myosin-I force sensitivity is also isoform dependent, and recent kinetic analysis shows that sequence differences in the motor can tune the force-sensitive mechanochemical transition (Rathbone and Houdusse, 2025).

At the membrane scale, tension is commonly reported in mN/m. A reconstituted membrane-cortex system reached a lower-bound lysis tension of approximately 0.14 mN/m, within the 0.3 to 5.0 mN/m range measured by other membrane-based methods (Sakamoto et al., 2023). In human induced pluripotent stem cells, elevated membrane tension increased Myo1E recruitment during clathrin-mediated endocytosis (Smith et al., 2026), but a universal numerical threshold for Myo1E recruitment has not been established. Tension values should therefore be interpreted within the measurement method and biological context.

Computational models further show threshold behavior. In the agent-based lamellipodial model of Kim and Kim (2025), continuous retrograde flow required a relative Arp2/3 density of at least 0.008 and a crosslinker density of at least 0.02. The reference model used a myosin transition rate of 17 s−1, corresponding to a walking speed of about 120 nm/s, and the dynamic steady state persisted for more than 200 s. These parameters are model specific, but they demonstrate how nucleation, network connectivity, motor kinetics, and adhesion resistance can be compared quantitatively.

Network architecture also changes how myosin-derived chemical energy is converted into mechanical work. In a purified system, the maximum instantaneous power was 4.0 ± 1.8 × 10−15 J/s in an Arp2/3-nucleated branched network compared with 1.5 ± 1.0 × 10−14 J/s without a nucleator. ATP consumption remained similar at 44.9 ± 11.4 and 47.4 ± 10.8 µM/min, respectively (Sakamoto and Murrell, 2024). These measurements show that high motor activity does not necessarily imply high mechanical work when branched network architecture constrains force transmission.

## 7 Regulatory context and experimental perturbation

Arp2/3-dependent actin assembly and myosin-dependent mechanics are coordinated by upstream signaling rather than by a single shared regulator. Rho-family GTPases, WASP/WAVE-family nucleation-promoting factors, mTOR signaling, post-translational modifications, and pharmacological perturbations provide complementary entry points into this regulation.

### 7.1 Rho family GTPases

Rho-family GTPases provide a major signaling link between protrusive and contractile actin systems. Rac1 and Cdc42 activate WAVE- and WASP-family pathways that stimulate Arp2/3, whereas RhoA activates ROCK and increases NMII regulatory light-chain phosphorylation (Sumida and Yamada, 2015; Lam and Chong, 2025). These pathways can be spatially separated and reciprocally regulated during migration and membrane remodeling.

During epithelial self-contact fusion, Rac1 and Cdc42 support Arp2/3-dependent actin assembly, while RhoA supports NMII-dependent contractility (Sumida and Yamada, 2015). The convergence of these pathways changes membrane apposition and adhesion organization. This is an example of signaling cross-talk between separate mechanical modules.

Oncogenic or epigenetic signaling can regulate protrusive actin assembly and ROCK–myosin-II contractility in parallel. In cancer cells, MLL1-dependent cytokine signaling coordinates an IL-6/8/pSTAT3/Arp3 axis with a TGF-β1/Gli2/ROCK1/2/pMLC2 axis, thereby influencing three-dimensional migration and metastasis (Nair et al., 2024). More generally, Rac–Arp2/3 and RhoA–myosin-II cross-talk can coordinate lamellipodial protrusion, intracellular pressure, and contractility (Patel et al., 2021).

### 7.2 mTOR signaling as an indirect regulatory context

mTOR signaling adds a regulatory layer that links membrane state, endolysosomal organization, and actin mechanics. Recent work demonstrated bidirectional codependence between mTORC1 activity and endolysosomal actin structures organized by WASH and Arp2/3 (Priya et al., 2023). This finding indicates that local branched actin is not simply a downstream output of mTORC1. In migrating cells, mTORC2 can act as a mechanochemical relay. Protrusion-associated increases in membrane tension activate an mTORC2-dependent pathway that stimulates distal Rho signaling and supports front–rear polarity (De Belly et al., 2026). These data place mTOR signaling within the regulatory network of actin mechanics in defined contexts, but they do not support a general model in which mTORC1 or mTORC2 directly activates Arp2/3.

The mTOR–actin relationship is particularly relevant to lysosomal dynamics, autophagy, and cancer. mTORC1 is organized on lysosomal membranes, where WASH–Arp2/3-dependent actin microdomains can influence endolysosomal organization (Priya et al., 2023). However, whether mTOR signaling directly coordinates Arp2/3-dependent actin assembly with myosin-dependent force generation remains unresolved. In invasive cancer cells, signaling programs can regulate Arp3-dependent protrusive assembly and ROCK–myosin contractility in parallel (Nair et al., 2024). The causal contribution of mTOR to this coordination during tumor invasion remains an important question for future study.

### 7.3 Actin arginylation as a network-level modifier

Actin arginylation modifies network organization rather than creating a demonstrated direct Arp2/3-myosin complex. Arginylated beta-actin is structurally conserved but shows altered engagement with myosin-II and changes the balance between Arp2/3-associated patches and formin-associated cables in the studied system (Pinto et al., 2026). The finding is therefore best interpreted as a network-level post-translational modifier whose consequences may depend on actin composition and cell type.

### 7.4 Pharmacological tools and interpretive limitations

CK-666 and CK-869 inhibit Arp2/3 through distinct allosteric mechanisms. CK-666 binds at the Arp2-Arp3 interface available in the inactive complex and stabilizes a conformation that cannot complete the activating short-pitch rearrangement (Hetrick et al., 2013). This conformational trapping does not require displacement of NPFs, ATP, actin monomers, or mother filaments.

CK-869 binds a hydrophobic pocket in Arp3 and reorganizes the sensor loop, thereby destabilizing the active short-pitch interface (Hetrick et al., 2013). The two compounds therefore converge on inhibition of activation through different binding sites. Mammalian Arp2/3 iso-complexes are not equally sensitive to either compound, so inhibitor phenotypes can depend on ARPC isoform composition (Cao et al., 2024).

These inhibitors also have important experimental limitations. CK-666 and CK-869 do not inhibit mammalian Arp2/3 iso-complexes equally, so phenotypes can depend on ARPC isoform composition (Cao et al., 2024). Blebbistatin is a broad myosin-II inhibitor and is problematic for live fluorescence imaging because blue-light exposure can cause photoinactivation and phototoxicity. Recent development of more selective NMII inhibitors also highlights the limited target selectivity and *in vivo* utility of blebbistatin (Radnai et al., 2025). These limitations should be considered when assigning a phenotype specifically to Arp2/3 or NMII activity.

Blebbistatin reduces NMII-dependent contractile output by inhibiting myosin-II ATPase activity, whereas newer NMII inhibitors offer improved selectivity and *in vivo* properties (Radnai et al., 2025). Parallel or combined perturbation of Arp2/3 and NMII can separate branched-actin assembly from contractility, but pharmacological co-dependence alone does not demonstrate direct molecular interaction. Conclusions should be supported by orthogonal genetic, imaging, or biophysical evidence whenever possible.

## 8 Disease relevance of disrupted Arp2/3–myosin regulation

Human disease shows that defects in branched-actin assembly or myosin-dependent mechanics can disrupt membrane-cytoskeleton function, but direct failure of Arp2/3-myosin coupling has been demonstrated only in selected settings. WASP-dependent actin regulation is central to immune-cell development and function (Lam and Chong, 2025). More direct evidence for Arp2/3 itself comes from ARPC5 deficiency: loss of ARPC5-containing complexes compromises macrophage phagocytosis and bacterial killing and causes inflammatory and immunodeficiency phenotypes (Vasconcellos et al., 2025). These findings establish causal disease relevance for branched-actin organization without proving that myosin coupling is the primary lesion.

MYH9-related disorders provide a complementary example on the contractile side of the system. Disease-associated defects in non-muscle myosin-IIA reduce platelet mechanical output and impair hemostasis (Baumann et al., 2022). These data do not indicate a primary Arp2/3 defect in MYH9-related disease. Instead, they demonstrate that membrane-associated cellular functions can fail when actomyosin force generation is insufficient, even when branched-actin nucleation is intact.

MYO6-associated deafness illustrates an isoform-specific disease mechanism. Cryo-EM analysis of myosin-VI identified deafness-associated variants at interfaces that regulate motor autoinhibition and activation (Niu et al., 2024). Because myosin-VI participates in endocytosis and membrane-tubule remodeling, altered motor regulation can disturb actin-dependent membrane traffic. Whether defective coordination with Arp2/3 is a primary cause of hearing loss, however, remains unresolved.

Cancer invasion can involve parallel dysregulation of branched-actin assembly and myosin contractility. In breast cancer models, MLL1-dependent cytokine signaling regulated Arp3-dependent actin assembly through an IL-6/8–STAT3 pathway and myosin contractility through a TGF-β1–Gli2–ROCK–pMLC2 pathway. Perturbation of this program reduced three-dimensional migration and metastatic burden (Nair et al., 2024). This is a disease-relevant example of coordinated regulation of protrusive and contractile modules. It should be classified as pathway-level coordination rather than direct Arp2/3–myosin binding.

## 9 Current controversies and unresolved questions

An unresolved issue is how cells allocate actin, membrane area, and regulatory proteins among Arp2/3-dependent branched networks and formin-dependent linear filaments. Contact-guidance studies on aligned collagen show reciprocal changes in pathway use (Foroozandehfar et al., 2025; Niraula et al., 2025), but they do not establish universal competition for a single limiting monomer pool. The balance is likely to depend on local signaling, capping activity, geometry, adhesion, and mechanical load.

A second question is whether myosin-generated tension directly promotes Arp2/3 branch disassembly. Branch removal is regulated by GMF, coronins, branch-junction age, and nucleotide state (Guo et al., 2018; Cao and Way, 2024; Ghasemi et al., 2024; Shatery Nejad et al., 2025). Defined loading can accelerate branch dissociation (Ghasemi et al., 2024), but current evidence does not establish a universal cellular mechanism in which NMII directly debranches Arp2/3 junctions. Myosin may instead alter turnover indirectly through network compression, flow, or accessory factors.

A third issue is how membrane tension changes Arp2/3-dependent actin assembly. Mammalian endocytic studies show that tension changes the requirement for myosin-I and the dynamics of Arp2/3 recruitment (Smith et al., 2026), while migration studies show that membrane tension can transmit information over cellular distances (De Belly et al., 2026). Whether tension primarily alters nucleation-promoting factor recruitment, Arp2/3 activation, filament elongation, or membrane coupling is likely to differ between endocytosis and migration.

Finally, many cells express several myosin isoforms within the same actin-rich compartment. Their coordination may involve spatial segregation, different load sensitivities, distinct cargo adaptors, or competition for actin tracks. Resolving these relationships will require simultaneous isoform-specific imaging and perturbation rather than measurements of total myosin activity.

### 9.1 Outlook and priorities for future work

Future work should prioritize four experimentally testable questions. First, simultaneous measurements of branch turnover, membrane tension, and isoform-specific myosin activity are needed in the same cell. Second, reconstitution should test whether myosin-generated load directly shortens Arp2/3 branch lifetime or acts mainly through network compression and accessory debranching factors. Third, mammalian endocytosis requires direct tests of whether myosin-I changes Arp2/3 recruitment through force sensing, scaffolding, or both. Finally, combined perturbation of Arp2/3, formins, and individual myosin isoforms is needed to determine how mixed actin architectures share limiting actin and transmit force.

## 10 Conclusion

Arp2/3 and myosin coordinate membrane–cytoskeleton remodeling through complementary rather than interchangeable functions. Arp2/3 nucleates branched actin networks, while polymerization of the resulting filaments generates protrusive or invaginating force. Myosin isoforms then couple actin to membranes, generate contractile tension, remodel network organization, or transport cargo according to their biochemical properties and cellular localization.

The molecular basis of this coordination varies across organisms and cellular contexts. Direct Arp2/3 activation by myosin-I is established in specific fungal systems, whereas mammalian myosin-I is better supported as a membrane–actin linker and load-sensitive motor. NMII usually acts through spatially separated contractile networks and signaling feedback, while myosin-VI contributes minus-end-directed transport and membrane-tubule remodeling. Mechanistic interpretation therefore requires explicit distinction among direct binding, mechanical coupling, signaling cross-talk, and correlated localization.

Future studies should combine reconstitution, quantitative live-cell imaging, force measurements, and optogenetic perturbation to measure branch lifetime, membrane tension, cortical flow, and isoform-specific myosin activity in the same system. Priorities include defining how mTOR and Rho signaling integrate protrusive and contractile modules, how Arp2/3 and formins share actin resources, and whether altered coordination is a causal disease mechanism rather than a secondary response. These approaches can provide a more testable framework for membrane–cytoskeleton mechanics in physiology and disease.
